# Integrative analysis of lncRNAs in rheumatoid arthritis: from bioinformatics to experimental validation

**DOI:** 10.1007/s10238-025-01589-z

**Published:** 2025-02-19

**Authors:** Ahmad Golestanifar, Arezo Masroor, Hengameh Khedri, Mohammadreza Saberiyan, Azim Nejatizadeh

**Affiliations:** 1 Molecular Medicine Research Center, Hormozgan Health Institute, Hormozgan University of Medical Sciences, Bandar Abbas, Iran; 2 Department of Medical Genetics, Faculty of Medicine, Hormozgan University of Medical Sciences, Bandar Abbas, Iran; 3 Endocrinology and Metabolism Research Center, Hormozgan University of Medical Sciences, Bandar Abbas, Iran

**Keywords:** Bioinformatics analysis, Systemic lupus erythematosus, SNHG15, OTUD6B-AS1, LINC00963, RNA-seq, lncRNA-miRNA-mRNA

## Abstract

Rheumatoid arthritis (RA) is a chronic autoimmune disease characterized by progressive joint damage and systemic inflammation. Despite advances in treatment, challenges persist in early diagnosis and personalized therapy. Long non-coding RNAs (lncRNAs) have emerged as pivotal regulators in immune pathways and inflammation, offering potential as diagnostic biomarkers and therapeutic targets. Using GEO datasets (GSE169082, GSE124373), we identified differentially expressed genes in peripheral blood mononuclear cells of RA patients. Functional enrichment and pathway analyses were conducted to elucidate their roles. Key lncRNAs (LINC00963, SNHG15, SNHG3) were experimentally validated via real-time PCR in patient samples. Protein–protein interaction networks and ceRNA networks were constructed to explore molecular interactions. Analysis revealed significant up-regulation of LINC00963, SNHG15, and SNHG3 in RA patients, correlating with inflammatory markers and immune cell profiles. ROC analysis demonstrated high diagnostic potential, particularly for SNHG3 (AUC: 84.3%). Pathway enrichment highlighted immune activation and disrupted autophagic processes. This study identifies novel lncRNAs with diagnostic and therapeutic potential in RA, emphasizing the integration of computational and experimental approaches. These findings lay the groundwork for precision medicine strategies to improve RA management.

**Supplementary Information:**

The online version contains supplementary material available at 10.1007/s10238-025-01589-z.

## Introduction

Rheumatoid arthritis (RA) is a chronic autoimmune disease primarily targeting the synovial joints, leading to progressive joint damage, pain, and disability. It affects approximately 1% of the global population, with a higher prevalence among women [1, 2]. The underlying pathogenesis of RA involves complex interactions between genetic predispositions, environmental triggers, and immune dysregulation, resulting in chronic inflammation and autoimmunity. Key features of RA include the infiltration of immune cells, production of pro-inflammatory cytokines, and activation of osteoclasts, which collectively contribute to joint destruction [3]. Despite advances in therapeutic approaches, challenges remain in early diagnosis and personalized treatment, necessitating the identification of reliable biomarkers and therapeutic targets to improve patient outcomes.

Non-coding RNAs, including long non-coding RNAs (lncRNAs) and microRNAs (miRNAs), have emerged as pivotal regulators of gene expression, playing crucial roles in diverse biological processes such as immune regulation, inflammation, and cellular differentiation [4]. Unlike coding RNAs, ncRNAs do not translate into proteins but act as key modulators in gene regulatory networks. lncRNAs, in particular, serve as molecular sponges for miRNAs, influencing gene expression through competing endogenous RNA (ceRNA) networks [5].

Recent studies have highlighted the dysregulation of lncRNAs and miRNAs in autoimmune diseases like RA [6]. These molecules can modulate immune responses and inflammatory pathways by interacting with protein-coding genes and signaling cascades. For instance, dysregulated lncRNAs have been implicated in processes such as apoptosis, innate immunity, and T-cell activation, all of which are central to RA pathogenesis [7]. Given their stability in biological samples and tissue-specific expression, lncRNAs and miRNAs hold great promise as diagnostic biomarkers and therapeutic targets in autoimmune disorders [8]. However, their precise roles in RA pathophysiology remain incompletely understood, warranting further investigation.

Bioinformatics analysis has revolutionized the study of complex diseases by enabling the identification of differentially expressed genes (DEGs), functional enrichment pathways, and molecular interaction networks. Despite the power of bioinformatics to predict regulatory networks, experimental validation remains indispensable for confirming these findings. This complementary approach bridges the gap between in silico predictions and biological reality, offering a comprehensive understanding of disease-associated molecular mechanisms. In RA research, this dual strategy is crucial for identifying reliable biomarkers and potential therapeutic targets, as computational insights alone may not fully capture the dynamic nature of gene regulation in autoimmune diseases.

While prior studies have investigated the role of lncRNAs in the pathogenesis of rheumatoid arthritis (RA), many have been limited to either bioinformatics analyses without experimental validation. This study stands out by integrating comprehensive bioinformatics analysis of multiple GEO datasets with experimental validation of key findings. Moreover, we systematically explore the potential diagnostic utility of novel lncRNAs in the context of RA, such as SNHG3, LINC00963, and SNHG15. By bridging computational predictions with biological validation, this work offers a robust framework for identifying novel lncRNA biomarkers and laying the groundwork for their potential therapeutic application in precision medicine.

## Materials and methods

### Data collection and identification of differential expressed genes

We queried the GEO database using the keywords “Rheumatoid Arthritis,” “Peripheral Blood Mononuclear Cells,” “Homo Sapiens,” and “RNA-seq” [9]. Based on the search results, two datasets were selected for gene expression analysis: GSE169082 and GSE124373. GSE169082 includes seven peripheral blood mononuclear cell (PBMC) samples, comprising four RA samples and three healthy control (HC) samples. GSE124373 provides non-coding RNA profiling data through microarray analysis, comparing PBMC samples from 28 RA patients and 18 HC.

Several measures were implemented to address potential biases arising from publicly available datasets, which may stem from variations in experimental design, sample processing, and data normalization. Datasets were selected based on clearly defined inclusion criteria, high-quality annotations, and standardized processing methods. Only datasets with comparable experimental conditions, such as those involving peripheral blood mononuclear cell (PBMC) samples, were included to minimize heterogeneity. For differential expression analysis, conservative statistical thresholds (log2 fold change ≥|1| and adjusted *P* value ≤ 0.05) were applied to reduce false positives and ensure robustness in identifying differentially expressed genes (DEGs). To further validate findings and overcome the limitations of computational predictions, experimental validation using real-time PCR was performed for key lncRNAs, including LINC00963, SNHG15, and SNHG3. This dual approach enhanced the reliability of the results. Additionally, functional enrichment and pathway analyses were conducted to confirm the biological relevance of the DEGs, ensuring consistency with known disease mechanisms and providing deeper insights into their functional roles.

To identify differentially expressed genes (DEGs), GEO2R was employed for analyzing gene expression differences between normal and RA samples. Differentially expressed lncRNAs (DELs) and mRNAs (DEMs) were filtered using a log2 fold change (log2FC) ≥|1| and an adjusted *P* value ≤ 0.05 as thresholds. Differentially expressed miRNAs (DEMIs) were defined with a p value ≤ 0.05. The differential gene expression analysis thresholds, including a log2 fold change (log2FC) ≥|1| and an adjusted *P* value ≤ 0.05, were selected to ensure a balance between biological significance and statistical robustness. A log2FC threshold of |1| corresponds to at least a twofold change in gene expression, which is widely recognized as biologically meaningful and sufficient to capture genes with substantial regulatory effects in disease conditions. The adjusted p value threshold of ≤ 0.05 was employed to minimize the false discovery rate (FDR) while maintaining statistical significance. These criteria have been frequently adopted in transcriptomic studies to identify differentially expressed genes with high confidence, enabling the detection of key molecular changes relevant to the pathogenesis of rheumatoid arthritis.

### Gene functional enrichment analysis

An online tool, DAVID (Database for Annotation, Visualization, and Integrated Discovery) version 2023q4, was employed to conduct functional enrichment analysis of Gene Ontology (GO) terms and pathway analysis using the Kyoto Encyclopedia of Genes and Genomes (KEGG) for the identified differentially expressed mRNAs (DEMs) [10].

### Integration of protein–protein interaction (PPI) networks and construction of a ceRNA network

We analyzed all mRNAs’ protein–protein interaction (PPI) network using the STRING database (http://stringdb.org/) with an interaction score threshold above 0.40 to identify hub genes [11]. The resulting PPI network was visualized in Cytoscape, where hub genes were determined using the Maximum Clique Centrality (MCC) algorithm available in the Cytohubba plugin [12]. Additionally, subnetworks within the PPI network were separately identified using the MCODE plugin in Cytoscape [13]. Furthermore, the StarBase database (version 2.0, https://starbase.sysu.edu.cn/) was employed to predict the miRNAs interacting with lncRNAs as well as the mRNA targets of these miRNAs [14].

### Sample collection, PBMC isolation, and RNA extraction

This study, approved by the Ethics Committee of Hormozgan University of Medical Sciences, involved the collection of 50 whole blood samples (25 from RA patients and 25 from HC) at Shahid Mohammadi Hospital, with informed consent obtained from all participants. PBMCs were isolated from 10 mL of blood using Ficoll density gradient centrifugation. Blood samples drawn into EDTA-coated tubes were diluted with PBS, layered over Ficoll solution, and centrifuged. The PBMC layer was carefully extracted, washed twice with PBS, and used for total RNA extraction. RNA was extracted using the TRIzol method with the RNX-PLUS kit, quantified by NanoDrop spectrophotometry, and its quality was confirmed by 2% agarose gel electrophoresis. The RNA was then stored at -80°C for further analysis.

### cDNA synthesis and quantitative PCR

Complementary DNA (cDNA) was synthesized from the extracted RNA using a cDNA synthesis kit (Sinaclon), ensuring equal RNA input for all samples. Quantitative PCR (qPCR) analysis was performed using a Mic qPCR system and a SYBR Green Master Mix (Ampliqon, Denmark). The expression of selected lncRNAs (*SNHG15*, *SNHG3*, and *LINC00963*) was verified by qPCR, using B2M as the internal reference gene. The primer sequences for *B2M* and the lncRNAs are listed in Table 1.

**Table 1:** Primer sequences of selected lncRNAs and housekeeping gene

Gene name	Forward primer (5′–3′)	Reverse primer (5′–3′)
B2M	GCAGCATCATGGAGGTTTGAAG	TCAAACATGGAGACAGCACTCA
SNHG3	GTAAGGTTTAAAAAGGATGCTTCGC	GTGCAGATCGTCTTCTCTCCAAATA
LINC00963	CTAAGAAAACTGCCCCACATCATC	CAGGTGTCACTGAAGTTTCGTAAG
SNHG15	GGTGACGGTCTCAAAGTGGA	GCCTCCCAGTTTCATGGACA

### Data visualization

This study employed multiple tools to enhance data visualization and manuscript preparation. Cytoscape was utilized to construct and analyze both protein–protein interaction (PPI) networks and the ceRNA network to visualize complex network interactions [15]. Cytoscape provided an efficient means of visualizing large datasets, enabling the clear presentation of key biological interactions. Additionally, SRPlot was used to generate various statistical plots, facilitating a comprehensive visualization of gene expression data and enrichment analysis results [16]. These tools significantly improved the interpretability of the findings and the quality of the manuscript.

### Statistical analysis

Statistical analysis was performed using GraphPad Prism v8.4.3 (GraphPad Software Inc., USA). A combination of statistical methods was employed to ensure robust data interpretation. Quantitative real-time PCR (qPCR) data were analyzed using the Pfaffl method, allowing for relative quantification of gene expression levels. The normality of the data was assessed using the Shapiro–Wilk test to determine the appropriate statistical tests. The Mann–Whitney U test, suitable for non-parametric data, was used to compare two independent groups. Correlation analysis was conducted using Spearman’s rank correlation coefficient, a statistical method appropriate for evaluating relationships in non-normally distributed datasets.

## Results

### Differentially expressed genes in the RA

**Fig. 1:**
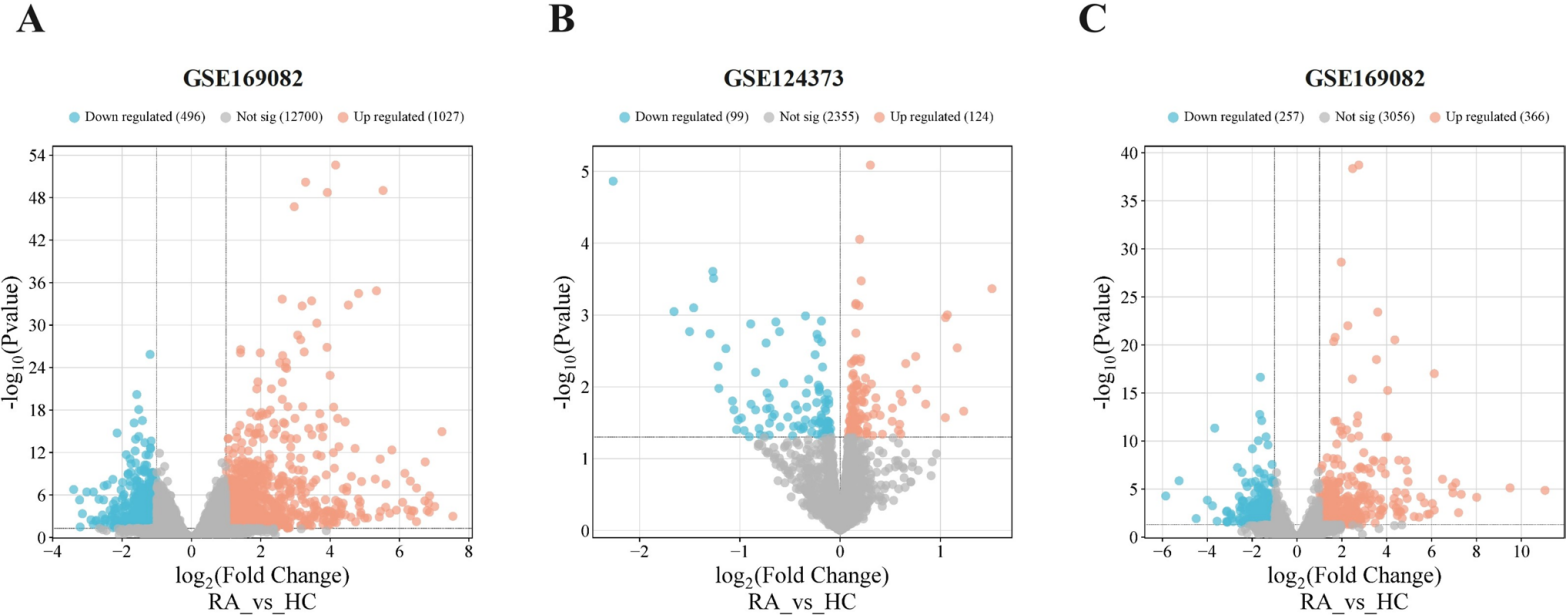
Distribution of differentially expressed genes (DEGs) in RA patients compared to HCs across datasets. **A** DEMs in GSE169082 **B** DEMIs in GSE124373, including 124 up-regulated and 99 down-regulated. **C** DELs in GSE169082 show 366 up-regulated and 257 down-regulated. Red and blue dots indicate up- and down-regulated DEGs, respectively, while gray dots represent non-significant changes (color figure online)

This study identified 1,523 mRNAs showing differential expression (DEMs) in PBMCs, highlighting significant differences between individuals with RA and HC. Among these, 1,027 mRNAs were found to be up-regulated, whereas 496 were down-regulated **(**Fig. 1A**)**. Additionally, an analysis of the GSE175840 dataset revealed 223 miRNAs with differential expression (DEMIs), comprising 124 up-regulated and 99 down-regulated miRNAs **(**Fig. 1B**)**. Moreover, 623 lncRNAs with altered expression (DELs) were discovered in PBMCs from RA patients compared to HCs. Of these, 366 exhibited significant up-regulation, and 257 were significantly down-regulated **(**Fig. 1C**)**.

### Functional enrichment analysis of

The functional enrichment analysis of differentially expressed mRNAs (DEMs) was conducted using the DAVID tool to explore their biological roles and cellular localizations. Gene Ontology (GO) enrichment analysis identified significant biological processes (BP), cellular components (CC), and molecular functions (MF) associated with these DEMs.

**Fig. 2:**
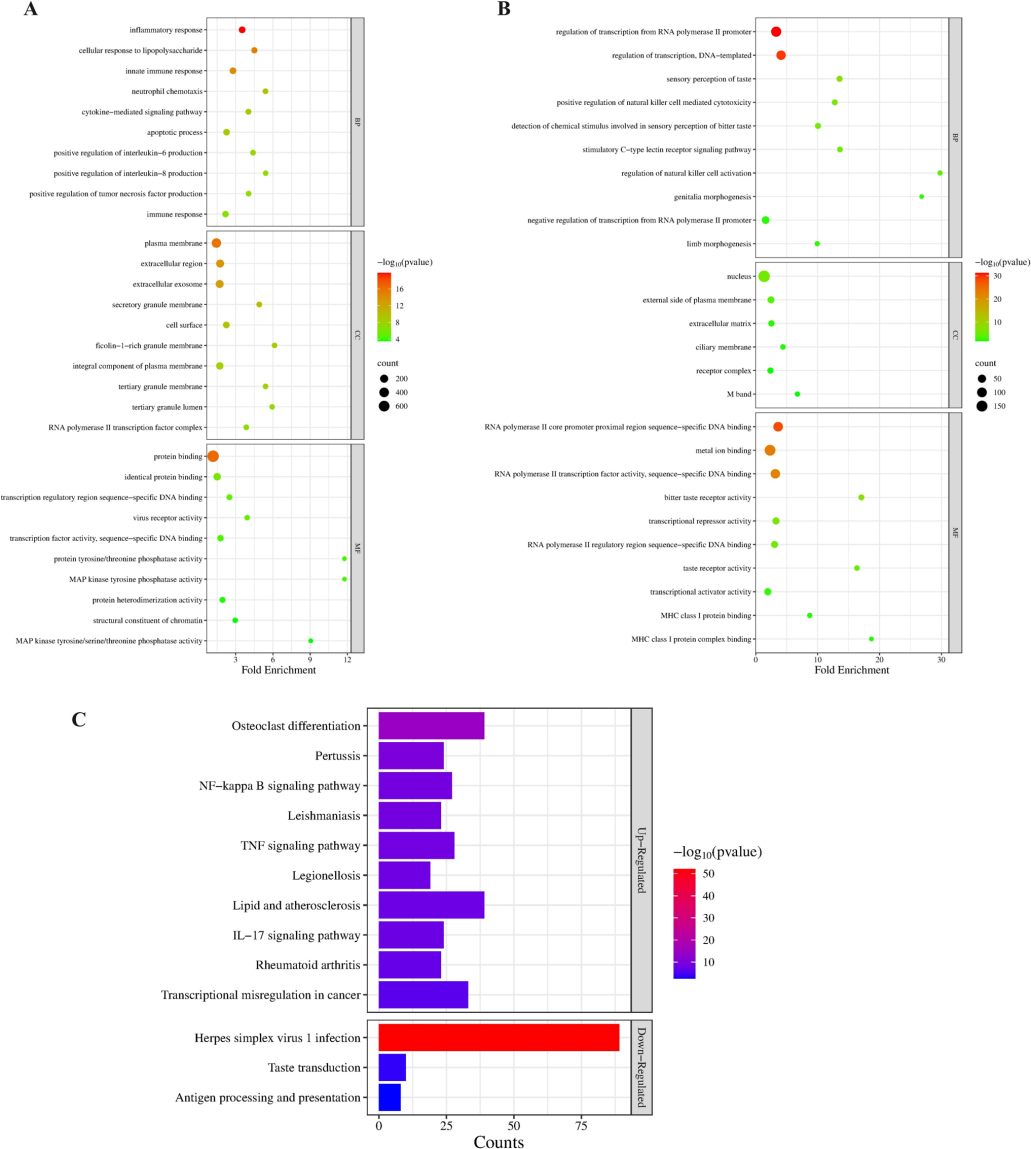
Functional enrichment analysis of DEMs in RA. **A** GO enrichment of up-regulated DEMs. **B** GO enrichment of down-regulated DEMs. **C** KEGG pathway analysis of DEMs. The size and color of the dots in (**A**, **B**) represent the number of genes and -log10(*P* value), respectively, while bar lengths in **C** reflect the gene count in each pathway

BP enrichment analysis showed that the up-regulated DEMs were significantly enriched in immune-related biological processes such as “innate immune response,” “inflammatory response,” and “apoptotic process.” These findings suggest that up-regulated DEMs are involved in activating immune pathways and cell death mechanisms in response to external stressors. In terms of cellular components, these DEMs were associated with “extracellular exosome,” “cytoplasm,” and “plasma membrane,” indicating their roles in extracellular communication and intracellular signaling. From the molecular function perspective, the top enriched functions included “protein binding,” “identical protein binding,” and “actin binding,” highlighting their involvement in forming protein complexes and cytoskeletal regulation **(**Fig. 2A**)**.

On the other hand, the down-regulated DEMs were primarily enriched in processes related to the “regulation of DNA-templated transcription,” “cell division,” and “DNA damage response.” This suggests a down-regulation of key DNA regulation and cellular replication pathways. The associated cellular components included “nucleus,” “nucleoplasm,” and “centrosome,” reflecting the role of these genes in the nuclear compartment and genetic material processing. In terms of molecular functions, enrichment was observed for “RNA polymerase II cis-regulatory region sequence-specific DNA binding” and “protein binding,” implying reduced activities in transcriptional regulation and general protein–protein interactions **(**Fig. 2B**)**.

The KEGG pathway enrichment analysis highlights significant changes in up-regulated and down-regulated pathways. Among the up-regulated pathways, key findings include enhanced “Osteoclast differentiation,” “Transcriptional misregulation in cancer,” and “NF-kappa B signaling pathway,” indicating increased cellular stress, immune responses, and transcriptional changes often linked to cancer. Other pathways such as “TNF signaling,” “Lipid and atherosclerosis,” and “IL-17 signaling” further emphasize active immune and inflammatory responses. On the other hand, down-regulated pathways prominently include “Herpes simplex virus 1 infection,” reflecting suppressed viral response activity, along with reduced activity in “Taste transduction” and “Antigen processing and presentation,” suggesting decreased sensory and immune processing mechanisms. These results underscore a shift toward heightened immune and inflammatory activity with suppressed viral and antigen processing functions **(**Fig. 2C**)**.

In summary, the up-regulated DEMs emphasize immune activation and extracellular signaling, while the down-regulated DEMs point to decreased DNA-related processes and transcriptional regulation. The KEGG pathway analysis further underscores these regulatory shifts, revealing critical changes in cellular stress, immune function, and disease-associated pathways.

### Identification of hub genes and network analysis

**Fig. 3:**
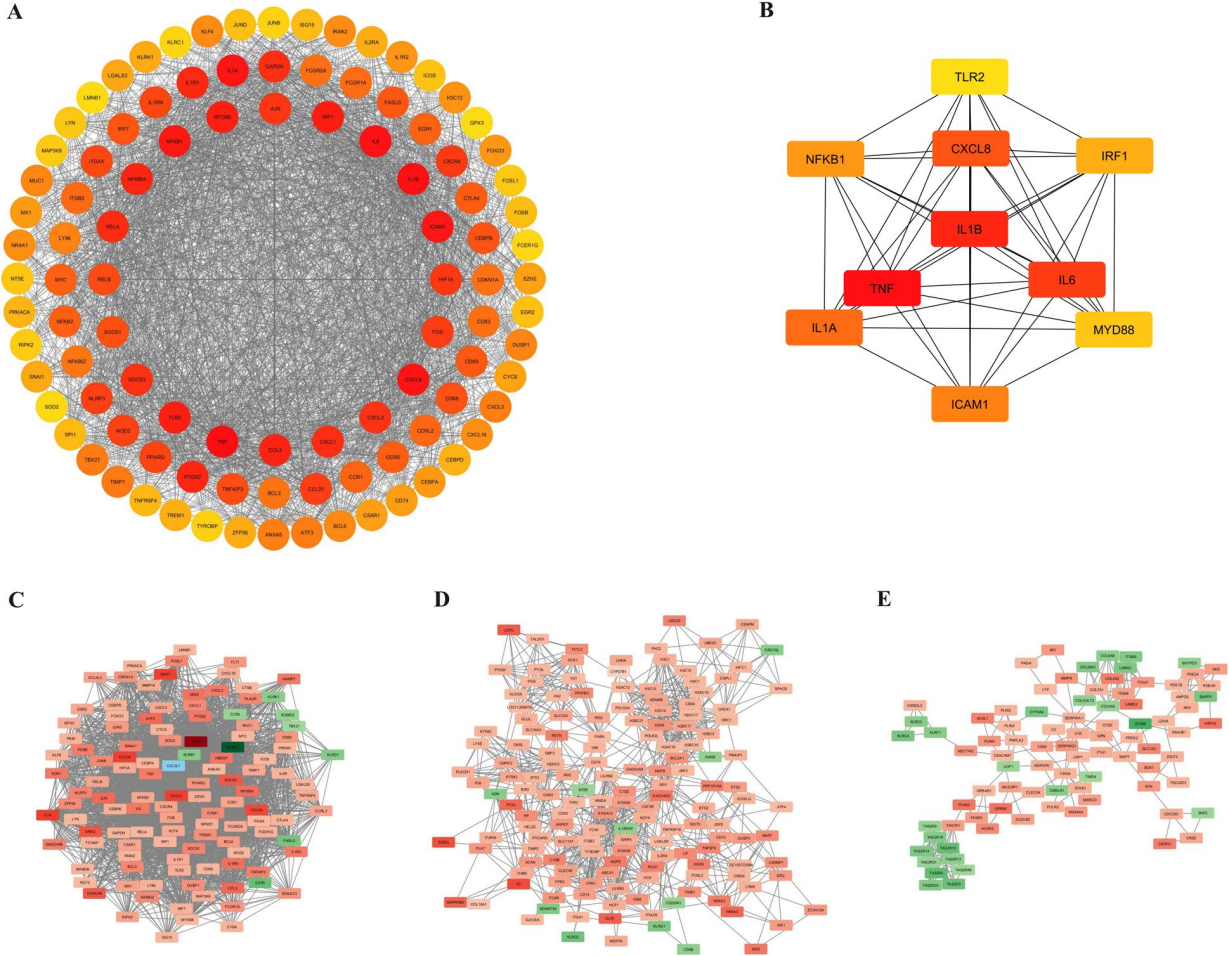
Protein–protein interaction (PPI) network and identification of hub genes in RA. (**A**, **B**) The top 100 and top 10 hub genes in the RA network were identified using the MCC method, respectively. Node size and color intensity represent the degree of connectivity, with red indicating higher centrality. (**C**–**E**) Key submodules of the PPI network were identified using the MCODE algorithm. Red and green colors represent up-regulation and down-regulation, respectively (color figure online)

A Protein–Protein Interaction (PPI) network was constructed using the STRING database to explore the interactions between proteins encoded by the DEMs. The network consisted of 1,317 nodes and 10,332 edges. The top 100 hub genes were selected for further analysis using the MCC method within the PPI network **(**Fig. 3A**)**. The top 10 hub genes were selected for regulatory network construction **(**Fig. 3B**)**. The expression data for these ten hub genes are provided in Table 2. Additionally, we identified significant modules within the PPI network using another Cytoscape plugin, Molecular Complex Detection (MCODE). The most important modules retrieved by MCODE are shown in Fig. 3C–E.

**Table 2:** Top 10 hub genes expression information

Symbol	Description	Gene type	EnsemblGeneID	log2FoldChange	Base mean	adjusted *P* value	*P* value
CXCL8	C-X-C motif chemokine ligand 8	protein-coding	ENSG00000169429	6.742709	4540.23	2.19E-11	1.59E-13
ICAM1	intercellular adhesion molecule 1	protein-coding	ENSG00000090339	3.211745	1421.78	3.33E-19	7.33E-22
IL1A	interleukin 1 alpha	protein-coding	ENSG00000115008	6.86603	206.79	0.000268	0.00002
IL1B	interleukin 1 beta	protein-coding	ENSG00000125538	7.226271	7726.72	1.14E-15	4.36E-18
IL6	interleukin 6	protein-coding	ENSG00000136244	4.07887	68.01	0.00513	0.000748
IRF1	interferon regulatory factor 1	protein-coding	ENSG00000125347	1.223163	6177.04	1.76E-05	7.84E-07
MYD88	MYD88 innate immune signal transduction adaptor	protein-coding	ENSG00000172936	1.134672	1966.14	5.78E-07	1.47E-08
NFKB1	nuclear factor kappa B subunit 1	protein-coding	ENSG00000109320	1.47783	3169.99	0.00217	0.000258
TLR2	toll like receptor 2	protein-coding	ENSG00000137462	1.549902	4201.61	2.22E-11	1.63E-13
TNF	tumor necrosis factor	protein-coding	ENSG00000232810	3.935682	755.13	1.63E-04	1.10E-05

### Regulatory network analysis

**Fig. 4:**
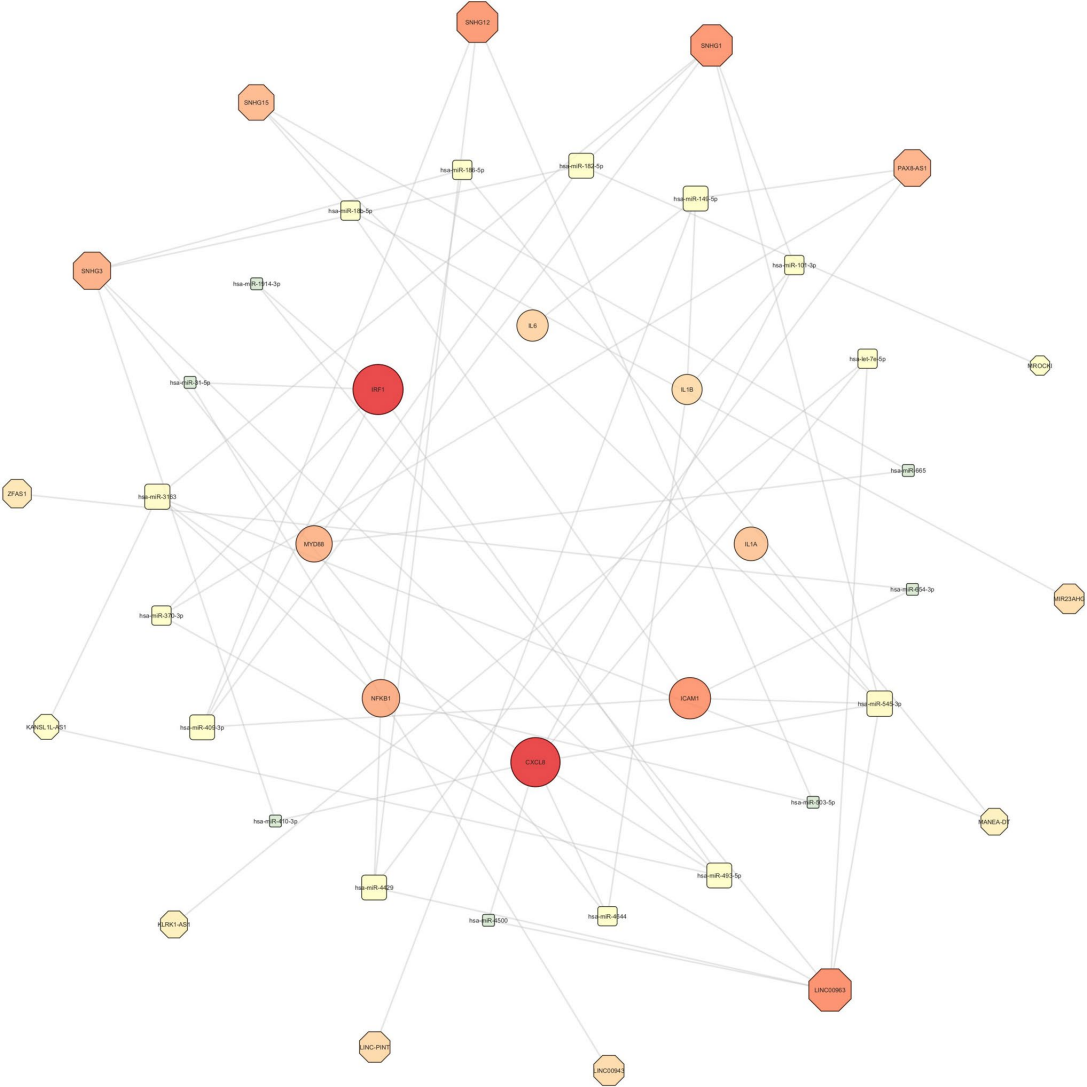
lncRNA-miRNA-mRNA regulatory network in RA. The network integrates lncRNAs, miRNAs, and mRNAs, highlighting their interactions. Octagonal nodes represent lncRNAs, square-shaped nodes represent miRNAs, and circular nodes represent mRNAs. Node size and color intensity correlate with higher interaction scores, emphasizing their central role in the network, with red indicating higher significance (color figure online)

The regulatory relationships between DELs, DEMIs, and the top 10 hub genes (CXCL8, ICAM1, IL1A, IL1B, IL6, IRF6, MYD88, NFKB1, TLR2, and TNF) were analyzed using the StarBase database. Each miRNA target of lncRNAs and each mRNA targeted by miRNAs were predicted. Subsequently, a lncRNA-miRNA-mRNA regulatory network was constructed by merging lncRNA-miRNA and miRNA-mRNA pairs while excluding nodes not connected to the main network (Fig. 4).

### Overexpression of *SNHG15*, *SNHG3 *, and* LINC00963* in RA

Based on the regulatory network constructed, randomly, three novel lncRNAs were selected for further investigation. The expression patterns of three novel lncRNAs (LINC00963, SNHG15, and SNHG3) were evaluated between RA patients and HC.

**Fig. 5:**
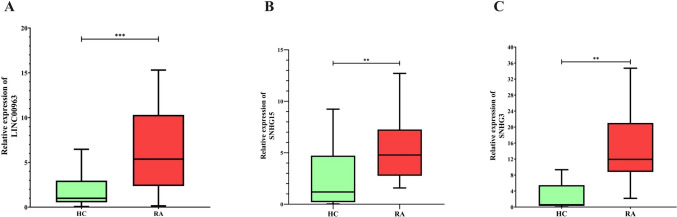
Relative expression levels of lncRNAs in RA patients compared to healthy controls (HC). **A** LINC00963 shows significantly higher expression in RA patients than HC, indicating its potential role in RA pathogenesis. **B** SNHG15 expression is significantly up-regulated in RA patients. **C** SNHG3 expression levels are markedly elevated in RA patients. Statistical significance: ***P < 0.001, **P < 0.01

The analysis of the expression levels of three lncRNAs, SNHG15, SNHG3, and LINC00963 in PBMCs from RA patients and HC demonstrates significant differences that suggest their potential involvement in the pathogenesis of RA. The expression of LINC00963 **(**Fig. 5A**)** was significantly higher in RA patients compared to HC, with a fold change of 4.09 (*P* < 0.001). This notable up-regulation suggests its critical role in inflammatory processes associated with RA. Similarly, SNHG15 **(**Fig. 5B**)** exhibited a significant increase in expression in RA patients compared to HC, with a fold change of 1.78 (*P* < 0.01), indicating its potential involvement in RA pathogenesis. Furthermore, SNHG3 **(**Fig. 5C**)** showed a pronounced up-regulation in RA patients, with a fold change of 2.54 (*P* < 0.01). These findings suggest a role for SNHG3 in the regulatory pathways contributing to RA.

Overall, the significant up-regulation of LINC00963, SNHG15, and SNHG3 in RA patients highlights their potential as key molecular players in the disease. These lncRNAs may serve as valuable biomarkers for RA diagnosis or as targets for therapeutic intervention. Further studies are necessary to explore their precise functions and interactions within RA-related molecular networks.

These findings suggest that LINC00963, SNHG15, and SNHG3 may be involved in the pathogenesis of RA, highlighting their potential as biomarkers or therapeutic targets.

**Table 3:** Summary of demographic and laboratory data, including blood cell counts, biochemical markers, and inflammatory indicators, from a study group. Values represent averages with corresponding ranges to highlight variability across participants

Variable	N	Average (range)
Age (years)	25	49.4 (24–76)
WBC (1000/μL)	21	7.3 (4.08–13.6)
RBC (Mil/Cumm)	21	4.89 (3.48–7.35)
Hemoglobin (g/dL)	21	11.76 (7–14.1)
Hematocrit (%)	21	37.16 (23.2–45.8)
MCV (fl)	21	75.60 (32.4–96.)
MCH (pg)	21	24.26 (16.71–31.3)
MCHC (g/dL)	21	31.41 (30.17–34.5)
RDW-CV (%)	21	16.07 (13.4–23)
Platelets (1000/μL)	21	283.14 (135–408)
MPV (fl)	14	10.93 (8.8–15.9)
PDW (%)	14	15.97 (15–17.1)
PCT (ml/L)	14	1.75 (0.223–4.18)
Neutrophils (%)	21	57.06 (39.9–79.2)
lymphocyte (%)	21	32.81 (12.2–50.4)
CRP (mg/l)	18	11.51 (0.67–63)
ESR (mm/hr)	18	29.05 (3–105)
Creatinine (mg/dL)	19	0.85 (0.63–1.19)
SGOT (IU/L)	18	24.77 (12–45)
SGPT (IU/L)	18	22 (11–38)
Ca	11	9.18 (7.9–10.6)
Ph	1	3.37 (2.6–3.8)

### Correlation of candidate lncRNA levels with characteristics of patients with RA

To explore whether the levels of SNHG15, SNHG3, and LINC00963 in PBMCs could serve as potential biomarkers for monitoring disease progression and overall health in patients with RA, we analyzed their correlation with RA patients’ characteristics (Table 3). Our analysis aimed to determine potential associations between lncRNA levels and disease severity, age, gender, and specific clinical markers, providing insights into lncRNA’s role in RA pathogenesis.

**Fig. 6:**
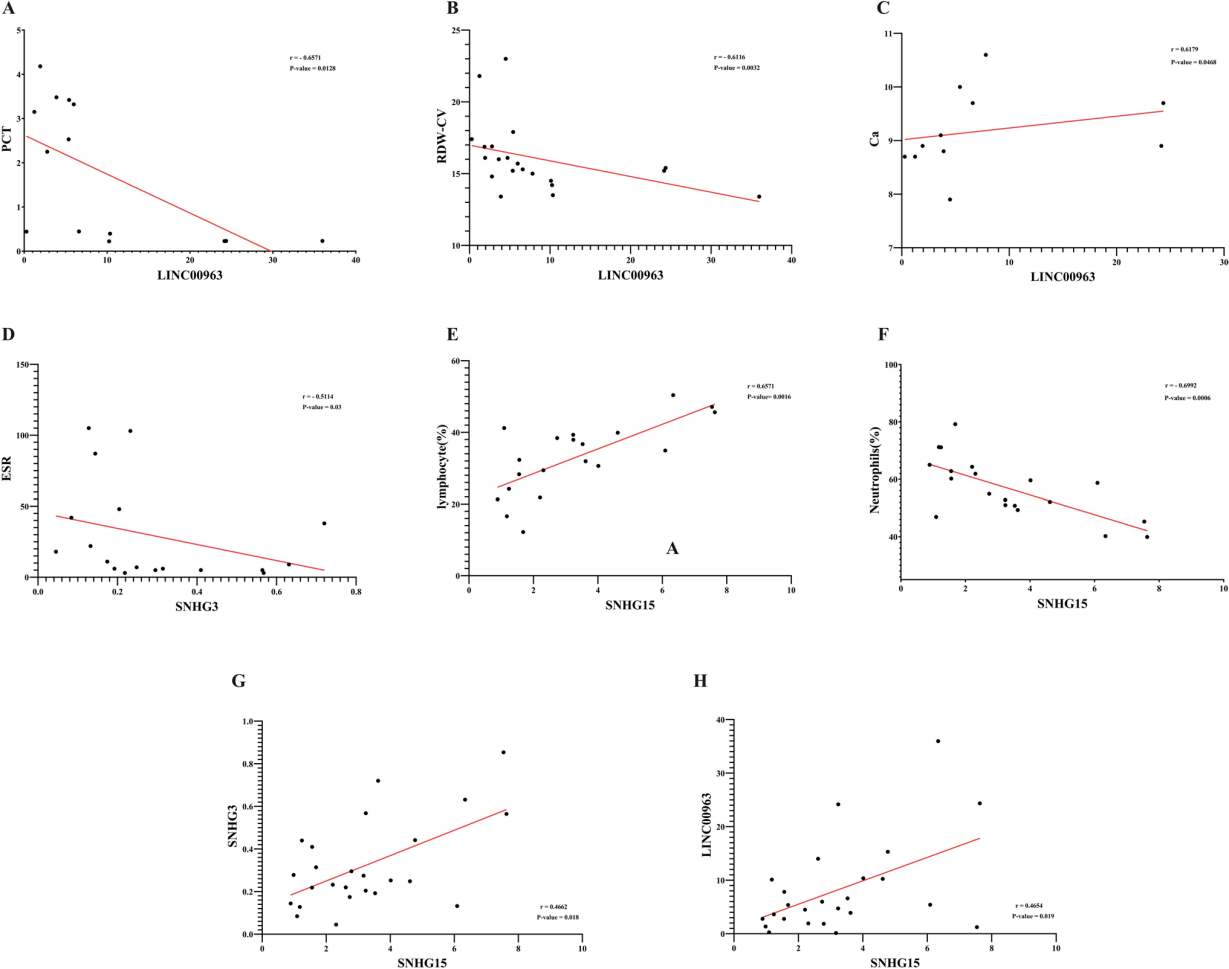
Correlation of lncRNA expression levels with clinical and hematological markers in RA patients. **A**–**C** LINC00963 exhibits significant negative correlations with PCT and RDW-CV and a positive correlation with calcium levels. **D** SNHG3 shows a significant negative correlation with ESR, suggesting its role in regulating inflammatory responses. **E** and **F** SNHG15 demonstrates a positive correlation with lymphocyte percentage and a negative correlation with neutrophil percentage, highlighting its role in immune cell balance. **G** and **H** Positive interrelationships are observed among lncRNAs, with SNHG15 correlating with SNHG3 and LINC00963, indicating potential shared regulatory pathways. The red trend lines represent linear regression for each correlation

LINC00963 displayed significant correlations with various inflammatory and hematological markers. Specifically, a strong negative correlation with plateletcrit (PCT) (r = − 0.6571, *P* = 0.0128) highlights its potential role in platelet-mediated inflammatory responses **(**Fig. 6A**)**. Additionally, a negative correlation with red blood cell distribution width (RDW-CV) (r = -0.6616, *P* = 0.0312) suggests its association with alterations in erythrocyte volume, often linked to systemic inflammation **(**Fig. 6B**)**. Furthermore, a strong positive correlation with calcium levels (r = 0.6179, *P* = 0.0468) indicates its involvement in metabolic or mineral balance disruptions frequently observed in chronic inflammatory conditions **(**Fig. 6C**)**.

SNHG3, despite its bioinformatics prediction of down-regulation, was experimentally found to be up-regulated in RA patients. This finding aligns with its observed inverse relationship with systemic inflammation, as indicated by a significant negative correlation with ESR (r = − 0.5114, *P* = 0.0301). This suggests that the up-regulation of SNHG3 might act as a compensatory mechanism to counterbalance inflammatory responses in RA pathophysiology. Furthermore, the positive correlation between SNHG3 and hematocrit (r = 0.4352, *P* = 0.0486) points to its potential role in maintaining erythropoietic balance or mitigating the effects of anemia of chronic disease, which is frequently observed in RA **(**Fig. 6D**)**. These findings underscore the complexity of SNHG3’s function, suggesting that it may contribute to both the regulation of inflammation and the stabilization of hematological parameters in RA.

SNHG15 displayed distinct relationships with immune cell proportions, emphasizing its role in immune regulation. A strong positive correlation with lymphocyte percentage (r = 0.6571, *P* = 0.0016) and a significant negative correlation with neutrophil percentage (r = − 0.6992, *P* = 0.0006) underline its association with adaptive immune responses and suppression of neutrophil-driven inflammation **(**Fig. 6E, F**)**. These findings suggest that SNHG15 may play a critical role in modulating the inflammatory microenvironment in RA.

Furthermore, interrelationships among these lncRNAs were evident. SNHG15 showed a positive correlation with LINC00963 (r = 0.4681, *P* = 0.0155) and a moderate association with SNHG3 (r = 0.4042, *P* = 0.042) **(**Fig. 6G, H**)**. These connections suggest shared or complementary roles in the regulatory networks influencing inflammation and immune responses in RA. The correlation details between the expression of each gene and the variables can be found in Supplementary File.

We also investigated the relationships between the expression levels of lncRNAs and other clinical characteristics (discontinuous variables), such as gender, rheumatoid factor (RF), and anti-cyclic citrullinated peptide (anti-CCP) antibody status. Although slight variations were observed, no differences reached statistical significance across clinical groups, indicating no strong associations between these expression levels and the clinical characteristics studied **(**Table 4**)**.

**Table 4:** Association analysis of lncRNAs expression levels with clinical characteristics in RA patients stratified by gender, RF, and anti-CCP status

	SNHG15	SNHG3	LINC00963
Gender			
Male	5.218	11.76	4.723
Female	3.878	13.45	8.465
P value	0.5110	0.7975	0.2154
RF			
Positive	3.2	10.35	5.355
Negative	2.773	17.29	5.39
*P* value	0.9229	0.2543	0.6744
Anti-CCP			
Positive	3.347	12.30	4.52
Negative	2.984	11.76	5.675
*P* value	0.8212	0.7283	0.5387

These findings highlight the differential roles of LINC00963, SNHG15, and SNHG3 in RA, with each lncRNA exhibiting unique but interconnected correlations with clinical and laboratory markers. These results suggest that these lncRNAs could be biomarkers for disease activity and potential therapeutic targets. Further studies are needed to elucidate their precise molecular mechanisms in RA pathogenesis.

### ROC curve analyses

**Fig. 7:**
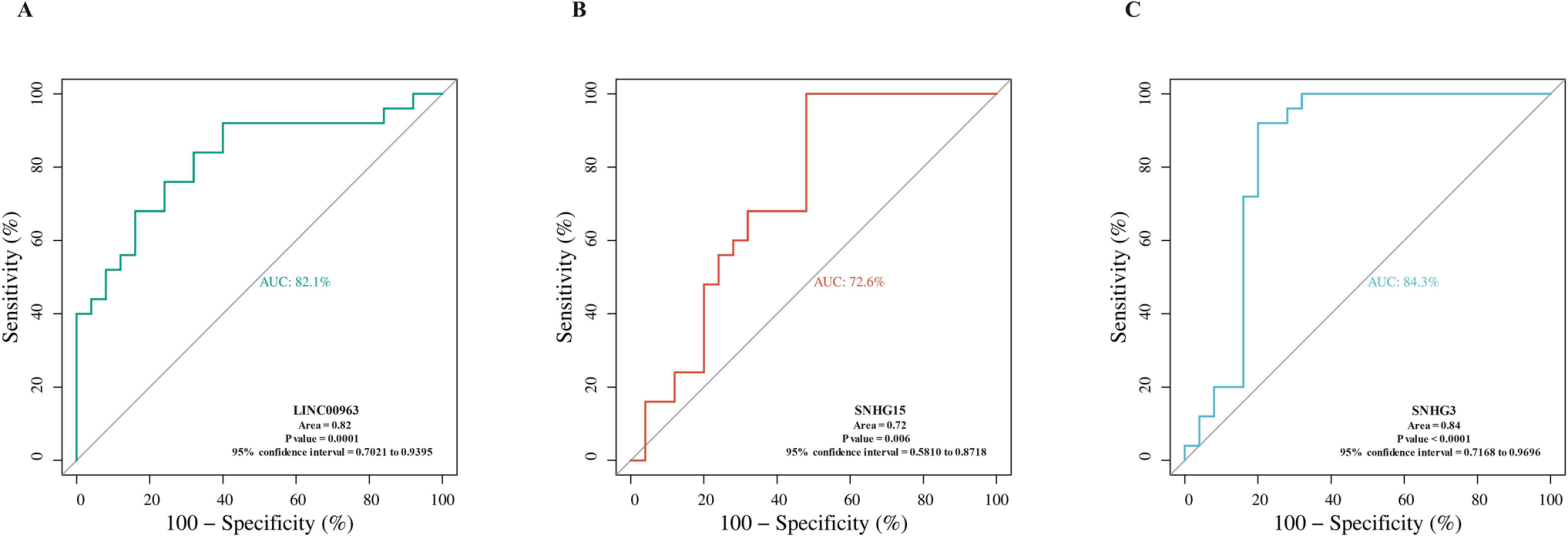
ROC curve analysis of lncRNAs for RA diagnosis. **A** LINC00963 shows a robust diagnostic performance with an AUC of 82.1% (*P* = 0.0001), highlighting its potential as a reliable biomarker for distinguishing RA patients from healthy controls. **B** SNHG15 exhibits moderate diagnostic capability with an AUC of 72.6% (*P* = 0.006), suggesting its role as a supplementary marker. **C** SNHG3 demonstrates the highest diagnostic accuracy with an AUC of 84.3% (*P* < 0.0001), underscoring its strong potential as a standalone diagnostic biomarker. Sensitivity and specificity values are indicated along the curve for each lncRNA

The ROC curve analysis for the lncRNAs LINC00963, SNHG3, and SNHG15 highlights their diagnostic potential in distinguishing RA patients from healthy controls. LINC00963 showed a robust diagnostic performance, with an AUC of 82.1% **(**Fig. 7A**)**, further supporting its utility in detecting RA. Among the three, SNHG3 demonstrated the highest diagnostic accuracy, with an area under the curve (AUC) of 84.3% **(**Fig. 7B**)**, indicating strong sensitivity and specificity as a biomarker for RA. In contrast, SNHG15 presented a relatively lower diagnostic performance, with an AUC of 72.6% **(**Fig. 7C**)**. While this indicates moderate diagnostic capability, it suggests that SNHG15 may be less effective as a standalone biomarker compared to SNHG3 and LINC00963. However, its significant correlation with immune cell dynamics and inflammatory markers highlights its potential role in complementing other biomarkers to provide a more comprehensive diagnostic profile.

These results collectively emphasize the strong diagnostic utility of SNHG3 and LINC00963 for RA, with SNHG3 emerging as the most reliable biomarker. Meanwhile, the moderate performance of SNHG15 suggests it may serve as a valuable auxiliary marker, particularly in cases where additional context about immune response and inflammation is needed. These findings support the integration of these lncRNAs into diagnostic workflows for RA, with further studies needed to validate their clinical applicability and explore their roles in disease progression and treatment response.

## Discussion

Rheumatoid arthritis is a complex autoimmune disease driven by a combination of immune dysregulation, chronic inflammation, and genetic predisposition, resulting in joint destruction and systemic complications [2]. While significant progress has been made in understanding the immune pathways involved in RA, there remains a critical need to explore non-coding RNA molecules due to their emerging roles in fine-tuning gene expression and modulating immune and inflammatory responses. Delving into the function and mechanisms of lncRNAs provides deeper insights into RA pathogenesis. It opens up new avenues for developing diagnostic biomarkers and innovative therapeutic strategies to mitigate disease progression and improve patient care.

This study provides a detailed analysis of DEGs, including mRNAs, lncRNAs, and miRNAs in RA, highlighting their potential roles in immune regulation and diagnostic applications. The integrative approach, combining bioinformatics predictions with experimental validation, offers valuable insights for advancing research and clinical strategies in autoimmune diseases.

Identifying 1,523 differentially expressed mRNAs (DEMs) and characterizing their functional enrichment emphasize the critical pathways involved in RA. Up-regulated DEMs were predominantly associated with immune activation and inflammatory responses, such as “innate immune response” and “apoptotic process.” These biological processes highlight the heightened immune system activity and cell death mechanisms that drive RA pathogenesis. Conversely, down-regulated DEMs were linked to fundamental processes like “regulation of DNA-templated transcription” and “cell division,” suggesting impaired genetic regulatory mechanisms. These findings align with known RA pathophysiology, where dysregulated immune pathways and genetic factors converge to sustain chronic inflammation.

Integration of protein–protein interaction (PPI) networks and identification of hub genes highlight pivotal molecular players in RA. Regulatory network analysis revealed that lncRNAs such as LINC00963, SNHG15, and SNHG3 may act as important factors influencing the interaction between mRNAs and miRNAs. LINC00963, SNHG15, and SNHG3 are lncRNAs that have garnered attention for their roles in cancer and autoimmune disorders. LINC00963 is primarily recognized for regulating immune responses and promoting cancer cell proliferation and migration through mechanisms like sponging miRNAs in ceRNA networks [17]. While research specifically linking LINC00963 to autoimmune conditions such as RA is limited, its expression patterns suggest a potential influence on inflammatory processes, positioning it as a promising candidate for diagnostic biomarkers or therapeutic interventions to modulate immune responses.

*SNHG15* (Small Nucleolar RNA Host Gene 15) is a well-studied oncogenic lncRNA that promotes cell proliferation, migration, and resistance to apoptosis in various cancers, including colorectal, breast, and lung cancer [18]. As a ceRNA, SNHG15 interacts with miRNAs to regulate key pathways like the MYC signaling pathway, which is vital in cell growth and division [19]. Its high expression levels correlate with advanced disease stages and poor prognosis in cancer patients [20]. These properties suggest its utility as both a diagnostic biomarker and a therapeutic target, with implications for modulating immune and inflammatory pathways in autoimmune disorders.

Similarly, SNHG3 has been linked to tumorigenesis and inflammatory processes, with growing evidence pointing to its role in promoting tumor growth and metastasis via interactions with regulatory miRNAs and proteins [21]. Though its specific functions in autoimmune diseases are less established, its involvement in inflammatory responses suggests a possible link to conditions like RA. Altered expression levels of SNHG3 may serve as diagnostic markers, while targeting this lncRNA could open avenues for new treatments addressing cancer and inflammatory diseases. LINC00963, SNHG15, and SNHG3 exemplify the potential of lncRNAs in expanding our understanding of disease mechanisms and offering novel diagnostic and therapeutic strategies.

The robust up-regulation of these lncRNAs in RA patients suggests their involvement in inflammatory cascades and immune dysregulation. The results of the expression analysis revealed that the experimental findings for SNHG15 and LINC00963 were consistent with the predictions from the bioinformatics study, indicating the up-regulation of these genes in RA patients compared to HCs. However, for SNHG3, there was a notable discrepancy between the bioinformatics prediction and the experimental results. Our study revealed a discrepancy between the bioinformatics analysis, which suggested down-regulation of SNHG3, and the Real-Time PCR results, which demonstrated significant up-regulation in RA patients. This inconsistency can be attributed to several factors that highlight the complexity of biological systems and methodological differences.

First, the difference in sample size between the RNA-Seq analysis (4 RA patients and 3 controls) and the Real-Time PCR study (25 RA patients and 25 controls) likely contributed to the conflicting results. As in the RNA-Seq analysis, a smaller sample size increases the risk of sampling bias and reduces the ability to capture the full heterogeneity of the patient population. In contrast, the larger sample size used in the Real-Time PCR analysis provides a more representative and statistically robust dataset.

Second, the heterogeneity of RA as a complex autoimmune disease might explain these observations. Diverse pathological mechanisms across patients characterize RA, and the RNA-Seq analysis, based on a small subset of individuals, may have captured a specific subpopulation or disease stage. In contrast, the larger cohort in the Real-Time PCR study will likely reflect a broader disease progression and patient variability spectrum.

Another possible explanation lies in SNHG3’s transcript specificity. RNA-Seq data may have been influenced by differential expression of a specific transcript of SNHG3, whereas Real-Time PCR primers might have targeted the overall expression of the gene, leading to an apparent increase in expression in RA patients.

Lastly, bioinformatics analyses were conducted on publicly available datasets, which may represent diverse patient populations with varying clinical characteristics, disease stages, or treatment histories. In contrast, our experimental cohort may have included a more homogeneous population with distinct inflammatory profiles, potentially influencing the expression levels of SNHG3.

To address these inconsistencies, future studies should consider increasing the sample size for RNA-Seq analysis to capture the variability within the RA population better. Additionally, using transcript-specific primers in Real-Time PCR experiments could help disentangle the contributions of different SNHG3 variants.

These results emphasize the importance of integrating in silico predictions with experimental validation to comprehensively understand the molecular mechanisms underlying RA pathogenesis.

The diagnostic potential of the analyzed lncRNAs is particularly noteworthy. ROC curve analyses demonstrated the utility of SNHG3 and LINC00963 as reliable biomarkers for RA, with SNHG3 emerging as the most sensitive and specific indicator. While SNHG15 exhibited moderate diagnostic accuracy, its correlations with immune cell dynamics suggest it may complement other biomarkers to provide a more nuanced diagnostic framework. These findings advocate for including these lncRNAs in diagnostic panels, paving the way for more accurate and early detection of RA.

Furthermore, the correlation of lncRNA expression levels with clinical and laboratory markers enriches our understanding of their roles in disease activity and immune regulation. For instance, SNHG15’s positive association with lymphocyte percentages and negative correlation with neutrophil percentages align with its role in adaptive immunity. At the same time, LINC00963’s relationship with inflammatory and metabolic markers indicates its involvement in broader inflammatory and metabolic disturbances. The compensatory up-regulation of SNHG3, as evidenced by its inverse correlation with ESR, suggests its role in mitigating inflammation, adding another dimension to its potential as a therapeutic target.

The implications of these findings extend beyond diagnostic and therapeutic applications. The observed interrelationships among the lncRNAs suggest they may function within interconnected regulatory networks, influencing multiple aspects of RA pathophysiology. This interconnectedness reinforces the need for systems biology approaches to unravel the complexity of autoimmune diseases.

While lncRNAs such as SNHG3, LINC00963, and SNHG15 show promise as novel diagnostic biomarkers for RA, several limitations must be acknowledged when comparing them to established clinical markers such as RF and anti-CCP antibodies. Established markers like anti-CCP antibodies exhibit high specificity for RA, making them reliable for confirming diagnoses. In contrast, while lncRNAs such as SNHG3 demonstrate strong diagnostic performance (e.g., AUC: 84.3%), their specificity and sensitivity in broader and more diverse patient populations remain underexplored. Rigorous validation in large, heterogeneous cohorts is necessary to confirm their diagnostic reliability.

In addition, lncRNAs often play roles in diverse biological processes and other diseases, unlike RF and anti-CCP, primarily associated with RA. For example, SNHG15 and LINC00963 have been implicated in cancer and other inflammatory conditions, raising concerns about their specificity for RA diagnosis. Incorporating lncRNAs into diagnostic workflows may require combining them with additional biomarkers to ensure disease-specific identification.

Despite these limitations, lncRNAs present unique advantages that could complement traditional markers. Their involvement in the early stages of RA pathogenesis may suggest the potential for earlier diagnosis compared to conventional markers that are often linked to established disease.

The diagnostic performance of lncRNAs as standalone markers may be limited compared to RF and anti-CCP. However, established markers like RF and anti-CCP are highly specific but may fail to identify seronegative RA patients, a subgroup comprising up to 20–30% of RA cases [22]. Sensitivity can be improved by incorporating lncRNAs, which are directly implicated in immune regulation and inflammation, into a diagnostic panel.

Integrating these lncRNAs into multi-marker panels could enhance diagnostic sensitivity and specificity. For instance, lncRNAs could provide additional insights into disease activity, severity, or progression, offering a broader diagnostic framework when combined with traditional markers.

Beyond diagnosis, lncRNAs such as SNHG3 and LINC00963 show correlations with disease severity and inflammatory markers, indicating their utility in monitoring disease progression and treatment response. Furthermore, incorporating lncRNAs into multi-marker diagnostic panels alongside traditional markers could enhance diagnostic accuracy and provide a more comprehensive assessment of disease activity and patient stratification.

Future research should focus on validating the clinical utility of lncRNAs through large, multi-center studies and developing standardized, cost-effective assays for their detection. Integrating these biomarkers into existing diagnostic workflows holds significant promise for advancing personalized medicine approaches in RA.

Despite these compelling findings, certain limitations must be acknowledged. The study’s reliance on public datasets for bioinformatics predictions may not fully account for population-specific genetic and environmental factors.

A notable limitation of this study is the relatively small sample size used for experimental validation. While sufficient to demonstrate initial findings, this sample size limits the generalizability and statistical power of the conclusions.

Expanding the sample size in future studies will enable more accurate stratification of RA patients based on clinical and demographic characteristics, such as disease severity, treatment history, and comorbidities. This will provide a clearer understanding of the diagnostic and prognostic potential of the identified lncRNAs.

Furthermore, larger cohorts would allow for subgroup analyses to explore the impact of age, sex, ethnicity, and genetic predisposition on lncRNA expression. The small sample size limits exploring dynamic changes in lncRNA expression during disease progression or treatment response. Longitudinal studies with larger patient populations could provide valuable insights into the temporal role of lncRNAs in RA pathogenesis and their potential as biomarkers for monitoring therapeutic efficacy.

Additionally, while experimental validation confirmed key observations, further functional studies are required to elucidate the precise molecular mechanisms and pathways influenced by these lncRNAs. Expanding the sample size and incorporating diverse populations will enhance the generalizability of these results.

## Conclusion

This study provides critical insights into the molecular mechanisms underlying RA by leveraging an integrative approach that combines bioinformatics predictions with experimental validation. Through the identification of differentially expressed mRNAs, lncRNAs, and miRNAs, the findings reveal their pivotal roles in immune regulation, inflammatory processes, and disease progression. Notably, the up-regulation of lncRNAs such as LINC00963, SNHG15, and SNHG3 underscores their significant involvement in inflammatory cascades and highlights their potential utility as biomarkers or therapeutic targets in autoimmune diseases.

The experimental validation of bioinformatics predictions using real-time PCR reinforces the reliability of the computational analyses, further establishing the diagnostic potential of these lncRNAs. By integrating computational and experimental methodologies, this work underscores the promise of systems biology approaches in unraveling the complexities of autoimmune pathophysiology.

In conclusion, this study contributes to identifying and validating potential diagnostic and therapeutic targets in RA. Integrating bioinformatics with experimental approaches provides a robust framework that paves the way for future research on improving clinical outcomes in autoimmune diseases.

## Supplementary Information

Below is the link to the electronic supplementary material.

Supplementary file1 (XLSX 13 KB)

## Data Availability

Available datasets: GSE162828 and GSE175840. These datasets are accessible through the Gene Expression Omnibus (GEO) repository https://www.ncbi.nlm.nih.gov/geo/.
